# 

**Published:** 1892-01

**Authors:** 


### CONTENTS:

ORIGINAL ARTICLES: PAGE

About Cattle Ticks. By Cooper Curtice, M.D. Vet. .... 1
Sterility of Mares. By M. E. Knowles, D.V.S. .... 7
Springhalt. By Williamson Bryden, V.S. .... 12
Parturient Apoplexy in Cows. By Dr. D. McIntosh .... 16
Parturient Apoplexy. By T. D. Hinebauch, M.S., V.S. .... 18
Influenza. By Mathew Wilson, M.R.C.V.S. .... 21
Purulent Metritis. By Dr. O. J. Lanigan .... 26
Reply to Dr. Peters. By Dr. D. E. Salmon .... 28
Clinic—Traumatic Tetanus .... 37
Recent Patents .... 38

SELECTIONS:

Royal Veterinary College .... 41
United States Veterinary Med. Asso'n, Comitis Minora .... 45
Worms in Sheep. By Edward Stanley, F R.C.V.S. .... 46
Experiments with Mallein. By E. E. Bennett, F.R.C.V.S., A.V.D. .... 53

SOCIETY PROCEEDINGS:

The Illinois Veterinary Medical Association .... 58
Massachusetts Veterinary Association .... 62
Montreal Veterinary Medical Association .... 63
Keystone Veterinary Medical Association .... 64

